# Testing multiplexed anti-ASFV CRISPR-Cas9 in reducing African swine fever virus

**DOI:** 10.1128/spectrum.02164-23

**Published:** 2024-04-02

**Authors:** Zezhong Zheng, Lei Xu, Yangbin Gao, Hongwei Dou, Yixuan Zhou, Xu Feng, Xiangjun He, Zhen Tian, Lingling Song, Guolong Mo, Jiapan Hu, Hongye Zhao, Hongjiang Wei, George M. Church, Luhan Yang

**Affiliations:** 1 South China Agricultural University, Guangzhou, China; 2 Qihan Biotechnology, Hangzhou, China; 3 Yunan Agriculture University, Kunming, China; 4 Harvard University, Cambridge, Massachusetts, USA; Oklahoma State University College of Veterinary Medicine, Stillwater, Oklahoma, USA

**Keywords:** African swine fever virus, CRISPR, pig, agriculture, xenotransplantation

## Abstract

African swine fever (ASF) is a highly fatal viral disease that poses a significant threat to domestic pigs and wild boars globally. In our study, we aimed to explore the potential of a multiplexed CRISPR-Cas system in suppressing ASFV replication and infection. By engineering CRISPR-Cas systems to target nine specific loci within the ASFV genome, we observed a substantial reduction in viral replication *in vitro*. This reduction was achieved through the concerted action of both Type II and Type III RNA polymerase-guided gRNA expression. To further evaluate its anti-viral function *in vivo*, we developed a pig strain expressing the multiplexable CRISPR-Cas-gRNA via germline genome editing. These transgenic pigs exhibited normal health with continuous expression of the CRISPR-Cas-gRNA system, and a subset displayed latent viral replication and delayed infection. However, the CRISPR-Cas9-engineered pigs did not exhibit a survival advantage upon exposure to ASFV. To our knowledge, this study represents the first instance of a living organism engineered via germline editing to assess resistance to ASFV infection using a CRISPR-Cas system. Our findings contribute valuable insights to guide the future design of enhanced viral immunity strategies.

**IMPORTANCE:**

ASFV is currently a devastating disease with no effective vaccine or treatment available. Our study introduces a multiplexed CRISPR-Cas system targeting nine specific loci in the ASFV genome. This innovative approach successfully inhibits ASFV replication *in vitro*, and we have successfully engineered pig strains to express this anti-ASFV CRISPR-Cas system constitutively. Despite not observing survival advantages in these transgenic pigs upon ASFV challenges, we did note a delay in infection in some cases. To the best of our knowledge, this study constitutes the first example of a germline-edited animal with an anti-virus CRISPR-Cas system. These findings contribute to the advancement of future anti-viral strategies and the optimization of viral immunity technologies.

## INTRODUCTION

African swine fever (ASF) is a viral disease that yields nearly 100% fatality rates in domestic pigs and wild boars globally (1, 2). The disease’s basic reproductive number (R0) is alarmingly high, up to 18.0, among domestic pigs (3). Over the past 4 years, outbreaks have emerged in 35 countries, presenting a global threat to the swine industry (4, 5). Advancements in ASF vaccinology, particularly the development of ASFV-G-ΔI177L/ΔLVR, BA71ΔCD2, and Lv17/WB/Rie1 vaccine strains, are promising to mitigate the disease’s socioeconomic and environmental impact on the global swine industry; however, challenges such as ensuring safety, cross-protection, and effective scale-up and commercialization still need to be addressed (6). Presently, the most impactful control measures still hinge on stringent quarantines (2).

Recent studies have highlighted the immense potential of utilizing CRISPR-Cas9 systems to target viral genomes, such as those of HBV and HIV, to manage viral titers *in vitro* by disrupting the vital genes or inactivating genome in a non-cleavage mechanism (7, 8). Targeting the ASFV p30 gene has shown significant potential in effectively suppressing viral replication. P30 protein plays a crucial role in the virus’s life cycle, as it is involved in host cell attachment and entry of ASFV (9). In our study, we explore the possibility of engineering a pig strain with acquired immunity against ASFV by incorporating an anti-ASFV CRISPR-Cas system into its genome.

## RESULTS

### Multiplex CRISPR strategy targeting the ASFV genome to protect pig cells from ASFV infection

**Fig 1:**
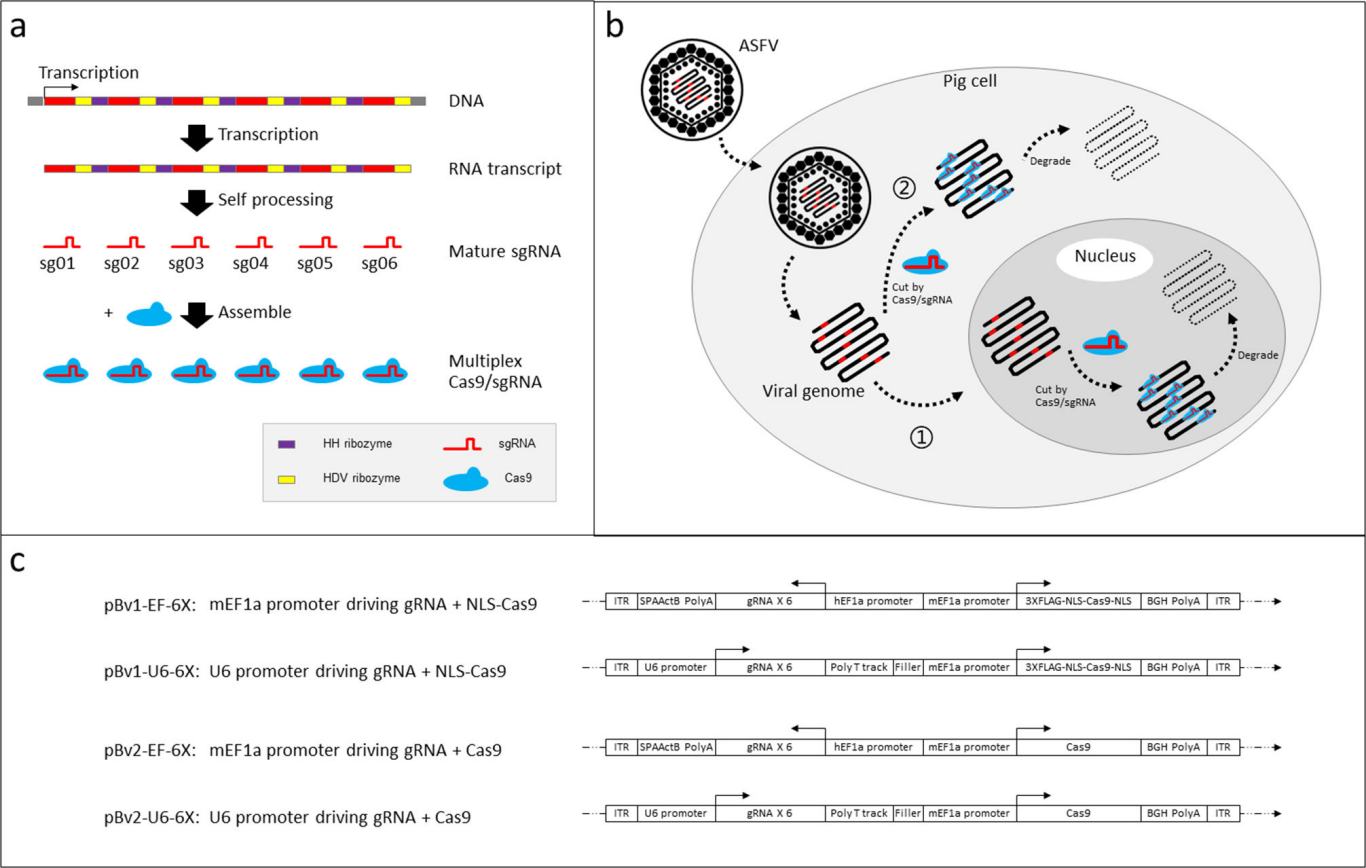
Multiplex CRISPR strategy targeting the ASFV genome to protect pig cells from ASFV infection. (a) DNA construct encoding six sgRNAs, each flanked by HH ribozyme and HDV ribozyme. Genes can be transcribed under one single promoter into a single RNA transcript that can be automatically processed into six mature sgRNA. The mature sgRNA assembles with Cas9 protein to form the catalytic active CRISPR complex. (**b)** sgRNA and Cas9 are constitutively expressed in the pig cells, cutting invading the ASFV genome either in the nucleus (route I) or cytoplasm (route II). The viral genome is subsequently degraded, and viral replication is stopped or attenuated. (**c)** Design illustration of the constructs for expressing both Cas9 protein and six sgRNAs. The transgenes are flanked by ITR sequences and inserted into the pig genome via *Piggybac* transposase. We tested Cas9 with and without the NLS signal. We also tested hEF1a and U6 promoter for 6-sgRNA expression.

ASFV is a double-stranded DNA virus with a genome size of 170,000 to 190,000 base pairs, encoding 150–200 proteins depending on the strain (10). We first analyzed all the published ASFV genomes and identified six guide RNAs (gRNAs), targeting nine places in the viral genome (Fig. 1a; Table S1). We hypothesized that by targeting multiple loci in the ASFV genome, we could minimize ASFV mutant escape. In addition, the cohort of targeting sites was chosen to cover the consensus sequences of all known ASFV strains and to avoid off-target cutting into the pig genome (Table S1). Next, we designed single transcription constructs to express these six gRNAs linked by ribozyme sites, driven by Type II and Type III RNA polymerase promoters, respectively (Fig. 1a and c, EF-1a and U6 promoters). It has been reported that the ASFV genome is exposed in both the cytoplasmic “virus factory” and nucleus, where it is under replication (11, 12) (Fig. 1b). As such, we designed Cas9-expressing constructs with or without nuclear localization signal (NLS) to test which one can achieve optimal targeting efficiency.

### Multiplex CRISPR strategy can efficiently cut ASFV DNA *in vitro* and different construct design has different effects on restricting ASFV replication in COS-7 cells

**Fig 2:**
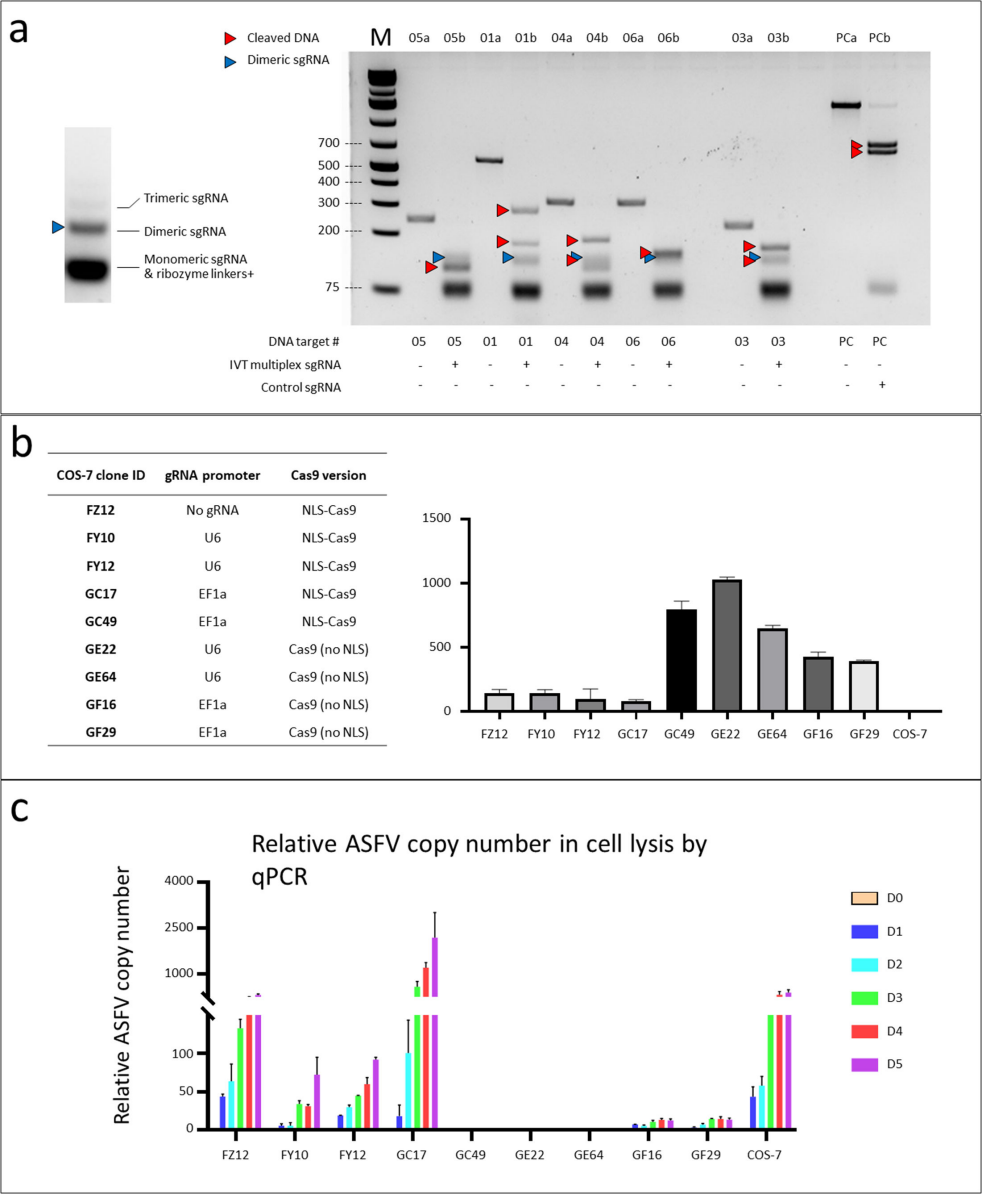
Multiplex CRISPR strategy can efficiently cut ASFV DNA *in vitro* and different construct design has different effects on restricting ASFV replication in COS-7 cells. (a) Left panel, the single RNA transcript generated by IVT can be efficiently processed into monomeric gRNA with few dimeric (blue triangle) and trimeric gRNA. Right panel, the IVT single RNA transcript can mediate efficient cleavage of PCR amplicons amplified from the ASFV genome in *vitro* Cas9 cleavage assay. “DNA target #” indicates the PCR amplicon corresponding to the respective gRNA. The starting PCR amplicons (lane 01a, 03a, 04a, 05a, 06a, PCa) are digested into two or more fragments (red triangles, lane 01b, 03b, 04b, 05b, 06b, PCb. PC: positive control) after co-incubation with IVT single RNA transcript and Cas9 protein. (**b)** Engineered COS-7 single-cell clones with different gRNA promoters and Cas9 versions (left table) and relative Cas9 expression levels in those clones (right panel) determined by RT-qPCR. (**)** ASFV replication in different COS-7 single-cell clones was measured by ASFV copy number in cell lysis using qPCR over 5 days. Strong inhibition of ASFV replication was only observed in COS-7 clones with high levels of Cas9 expression (GC49, GE22, GE64).

We first investigated the cutting efficacy of the CRISPR-Cas on the ASFV genome. To this end, we generated single gRNA transcripts via *in vitro* transcription (IVT) and observed robust cutting activities against ASFV genomic sequences (Fig. 2a; Fig. S2; Table S2). Later, we aimed to examine whether such a biochemical property of CRISPR-Cas can inhibit viral infection and replication. We chose COS-7 as the model system because it is permissive to ASFV infection and has been generally used as an *in vitro* model to quantify ASFV infectivity (13). We integrated the designed CRISPR-Cas constructs into the COS-7 genome via PiggyBac-transposition. Single-cell clones with CRISPR-Cas-gRNA integration were isolated, and corresponding Cas9 expressions were measured by RT-qPCR (Fig. 2b). Subsequently, we inoculated ASFV into the COS7 clones and monitored ASFV titers over 5 days. Interestingly, ASFV replication was significantly suppressed in the clones with high Cas9 expression (Fig. 2b, GC49, GE22, GE64, GF16, GF29 clones) but not in those with low Cas9 expression (FZ2, FY0, FY2, GC7 clones). This suggested that CRISPR-Cas inhibited ASFV replication in a dose-dependent manner (Fig. 2c). In addition, we found that the regulatory elements in the designed constructs were not determining factors for inhibiting ASFV replication. (i) Both Type II and Type III RNA polymerases were sufficient to drive the multiplexable gRNA expression, as evidenced by the significant inhibition of viral replication in Clone GE and GF. (ii) Cas9 with and without NLS could both significantly inhibit ASFV replication (Fig. 2, GE22, GF64 with NLS, GC49 without NLS). This observation is consistent with previous reports that ASFV utilizes both the host nucleus and cytoplasmic “virus factory” to replicate its DNA so that its genome is exposed in both sites (14, 15). Taken together, the data demonstrate that the multiplexable CRISPR-Cas9 can cut ASFV by cutting its genome at multiple loci and effectively prevent ASFV replication *in vitro*.

Having validated that CRISPR-Cas9 could target ASFV and inhibit its replication *in vitro*, we attempted to incorporate the construct into a pig strain and test if the resulting pigs gained immunity against ASFV infection. We chose the NLS-Cas9-EF1a-gRNA construct (identical to the construct of the GC49 COS7 clone) as it demonstrated significant ASFV suppression *in vitro* (Fig. 2c).

### Production of transgenic pigs constitutively expressing Cas9 and 6 sgRNA targeting ASFV genome and virus challenge of the transgenic pigs

**Fig 3:**
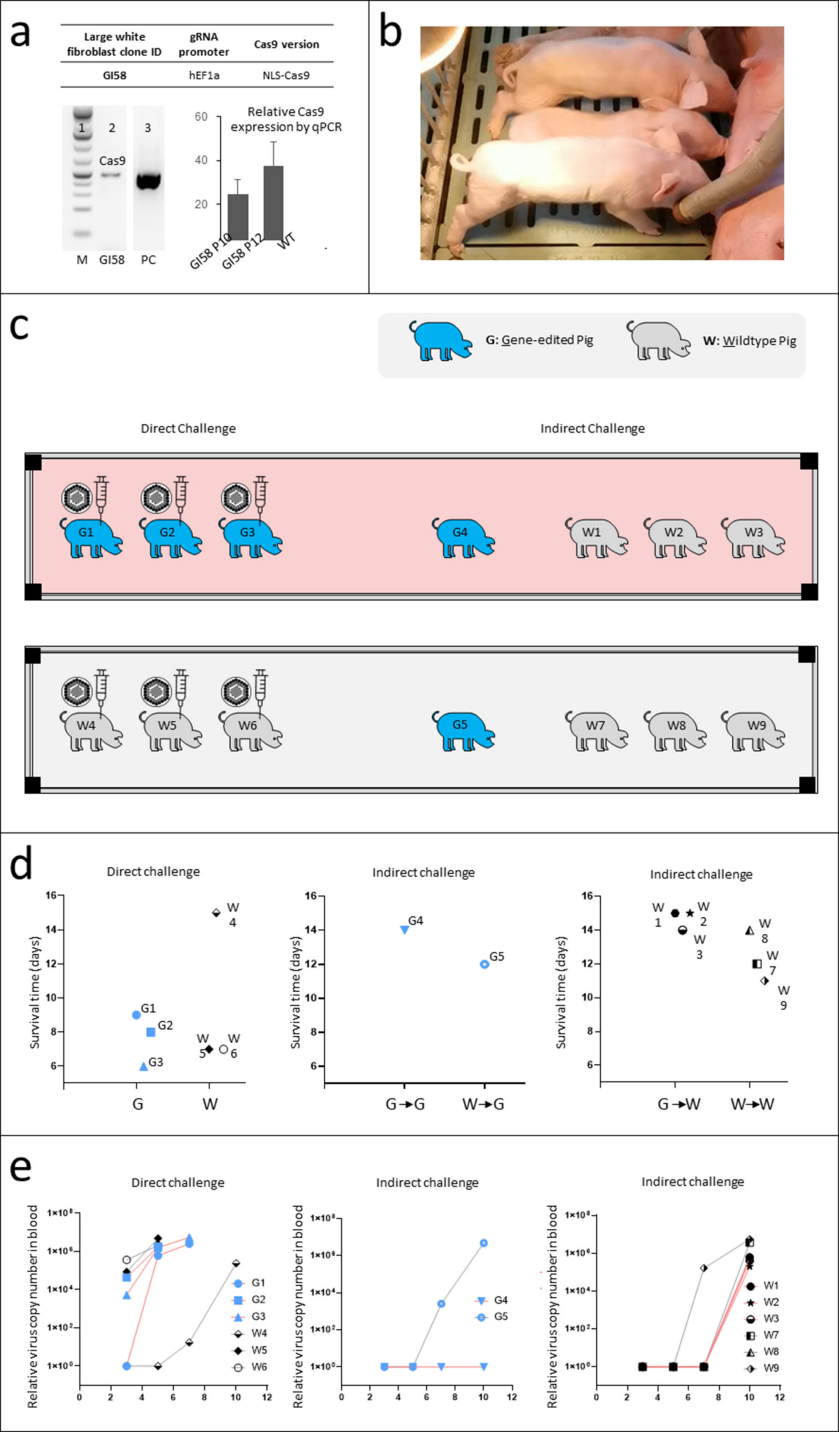
Production of transgenic pigs constitutively expressing Cas9 and 6 sgRNA targeting ASFV genome and virus challenge of the transgenic pigs. (a) Top table, the large white pig fibroblast single-cell clone GI58 harbors the pBv1-EF-6X construct with hEF1a promoter driving sgRNA and mEF1a promoter driving Cas9 with NLS. Bottom left panel, the presence of the transgene cassette was confirmed by PCR for the Cas9 gene in the genomic DNA of GI58. Bottom right panel, Cas9 expression was confirmed by RT-qPCR in the fibroblasts of the cloned pigs (GI58P10 and GI58P12) generated from GI58 fibroblasts. (**b)** photo of the cloned pigs generated from GI58 fibroblasts. (**c)** ASFV virus challenge design of the gene-edited pigs and wild-type pigs. Direct virus challenge was performed by muscular injection, while indirect virus challenge was performed by the cohabitation of the pigs in the same room with pigs under direct virus challenge. Each rectangle with four black squares indicates a separate room. In the light-red room, three gene-edited pigs were subjected to direct virus challenge, while in the light-gray room, three wild-type pigs were subjected to direct virus challenge. The additional one gene-edited pig and three wild-type pigs in each room are subjected to an indirect virus challenge. (**d)** Survival of the pigs in direct and indirect virus challenge. No statistical difference in survival time was observed in the two virus challenge modes comparing gene-edited pigs with wild-type pigs under the same condition. The arrow indicates indirect virus challenge, for example, W→G means wild-type pigs were under direct virus challenge and gene-edited pigs were under indirect virus challenge released by infected wildtype pigs. (**e)** ASFV titer in blood samples of the pigs in direct and indirect virus challenge measured by qPCR. Gene-edited pigs showed no detectable viral titer, much lower than wild-type pigs, in an indirect virus challenge released by infected gene-edited pigs (middle panel). No difference was observed in other conditions.

We first integrated the construct via the PiggyBac transposon system into the fibroblasts of a Large White pig strain. After isolating the single-cell clones with the validated genetic modification, we performed pig cloning via somatic cell nuclear transfer technology (SCNT) and successfully obtained pigs with the intended modifications (Fig. 3b and Materials and Methods). We validated genomic integration and the expression of Cas9 using the ear pouch sample isolated from the engineered pigs (Fig. 3a, GI58 P10, GI58 P12).

Next, we aimed to determine whether the newly established pig strain, genetically modified with multiplexed anti-ASFV CRISPR-Cas, could develop immunity against ASFV. We conducted this experiment using two separate challenge methods, direct and indirect and closely tracked the viral titers over time. In the direct challenge group, we inoculated an identical amount of ASFV intramuscularly into three transgenic pigs (G1, G2, and G3) and three wild-type pigs (W4, W5, and W6). The two groups were kept in separate housing to prevent cross-contamination. To assess immunity against ASFV through horizontal transmission, we also set up sentinel pigs groups. In this arrangement, one transgenic pig and three wild-type pigs, none of which had prior contact with ASFV, were placed in each of the two housing units. In the first, transgenic pig G4 and wild-type pigs W1, W2, and W3 shared quarters with the directly challenged transgenic pigs G1, G2, and G3. In the second unit, transgenic pig G5 and wild-type pigs W7, W8, and W9 cohabitated with the directly challenged wild-type pigs W4, W5, and W6. Throughout the course of the study, we maintained rigorous monitoring of each pig’s serum viral titers and survival time.

Consistent with previous reports (16, 17), intramuscular injection of ASFV was lethal and two wild-type pigs died within 7 days, although surprisingly, one outlier (W4) survived over 2 weeks with elevated ASFV titers in the blood (Fig. 3e). As expected, the pigs challenged indirectly survived longer (13.5 days vs 9.7 days) than directly challenged groups.

Interestingly, we observed no significant survival advantage of the transgenic pigs compared to the wild-type pigs in direct exposure groups (Fig. 3d, G1, G2, and G3 vs W4, W5, W6). This indicated that CRISPR-Cas was not sufficient to prevent ASFV replication *in vivo*. However, pigs cohabitating with infected transgenic pigs had a consistent extension in survival time (Fig. 3d and G4 vs G5; W1, W2, W3, vs W4, W5, W6), suggesting a potential delay in virus spread due to the CRISPR-Cas system. Notably, G4, the only transgenic pig that cohabitated with the infected transgenic pigs, demonstrated a significant delay in viral titer elevation (Fig. 3e). We reasoned that this could be attributed to the combination of the anti-ASFV CRISPR-Cas genome modification and the slower virus spread due to the effect of the CRISPR-Cas system in its cohabitants. However, by day 15, both wild-type and transgenic pigs succumbed to viral infection, highlighting the need for further optimization of the current strategy to combat ASFV *in vivo* (Fig. 3d).

## DISCUSSION

In summary, we developed a CRISPR-Cas9 system targeting nine genomic loci within the ASFV genome. We demonstrated that these constructs effectively disrupt the ASFV genome and manage viral titers *in vitro*. Interestingly, we discovered that both Type II (EF-1a) and III (U6) RNA polymerases, in addition to Cas9 with or without NLS, can achieve substantial ASF viral control *in vitro*. Moreover, we successfully bred a pig strain expressing this multiplexed anti-ASFV CRISPR-Cas system through germline genome editing. We observed a delayed onset of ASF symptoms in the transgenic herd, suggesting that pigs carrying the multiplexed CRISPR-Cas9 modification might exhibit some degree of resistance to ASF. However, transgenic pigs did not significantly outlive their wild-type counterparts upon direct ASFV challenges. Further investigations to refine the gene editing system and conduct comprehensive cohort studies are needed to clarify the effects of multiplexed anti-ASFV CRISPR-Cas9 and determine its potential for application in agricultural and clinical contexts.

To the best of our knowledge, our study presents the first example of an animal with an anti-virus CRISPR element in its genome generated via germline editing. Prior studies showed successful suppression of ASFV replication *in vitro* by targeting a single ASFV genome site (the CP204L gene) with CRISPR-Cas-gRNA in cell lines (9); however, it remains unknown whether such a mechanism works *in vivo*. In addition, targeting a single genomic locus is vulnerable to escape mutants; hence, a multiplexable gene editing approach is promising to eliminate the virus before accumulating mutants circumvent the acquired defense.

Encouragingly, we observed that pigs cohabitating with infected transgenic pigs exhibited a delay in ASFV-related death. Also, one transgenic pig that lived with other infected transgenic pigs had significantly lower serum viral titers, suggesting CRISPR-Cas could slow the virus’s spread within the herd. Nevertheless, we did not observe a significant survival advantage for transgenic pigs facing ASFV challenges. This might be due to the exceptionally high viral titers in our experiment, far exceeding those in typical contact-based infections. In addition, the expression level of Cas9-gRNA in the pigs might not have been sufficient. As suggested by our *in vitro* assays, the Cas9-gRNA expression level is inversely proportional to the extent of viral replication (Fig. 2b), so a higher expression level could potentially enhance immunity against ASFV. In the future, advanced genome-engineering work could be done to improve herd immunity in pigs, including increasing Cas9 and gRNA expression levels, delivering both Cas9 constructs with and without NLS to maximize the odds of cutting the viral genome throughout its life cycle (Fig. 1b and 2c), and using synthetic approaches to drive Cas9 expression in a tissue-specific or pathogen-induced manner.

Preventing the spread of ASFV is not just crucial for agriculture but also carries significant clinical implications. The recent advances in xenotransplantation have encouraged more clinical trials involving pig organ transplants in human patients (18, 19). However, this also necessitates stringent monitoring and control of zoonotic diseases transmitted by endogenous pig viruses like PERVs (20, 21) or exogenous porcine viruses such as ASFV and pig CMV. As we approach the end of the COVID-19 pandemic, it is imperative to preempt any potential cross-species viral transmission via pig organs to ensure the safety of clinical applications (22).

## MATERIALS AND METHODS

### CRISPR–Cas9 gRNA design

R library DECIPHER was used to design specific gRNAs (Table S2). Guide RNAs are designed to target the ASFV genome based on the BA71V strain (RefSeq: GCF_000858485.1). The potential gRNA off-targets in a large white pig genome are predicted bioinformatically. Select six sgRNAs with a lower probability of off-target effects.

### Plasmid construction

pBv1-EF-6X, pBv1-U6-6X, pBv2-EF-6X, and pBv2-U6-6X (Fig. 1c) were constructed by Golden Gate Assembly (NEB Golden Gate Enzyme Mix (BsaI-HF2), New England BioLabs) with one of the four backbone plasmids pBv1-EF-BB/pBv1-U6-BB/pBv2-EF-BB/pBv2-U6-BB and all the six TOPO-RGR (RGR: ribozyme-sgRNA-ribozyme) plasmids following the manufacturer’s instructions. The backbone plasmids were constructed by Gibson Assembly and NLS-Cas9/Cas9 was PCR amplified from the pX330-U6-Chimeric_BB-CBh-hSpCas9 vector (23). Each of the TOPO-RGR fragments was constructed by overlapping PCR followed by TOPO cloning using pClone007 Versatile Simple Vector Kit (Tsingke). The sequences of the multiplex sgRNA designs using either RNA pol II mEF1a promoter or U6 promoter are illustrated in Fig. S1.

### ASF virus strain

The gz201801 strain was isolated by Zhang et al. from South China Agricultural University. The virus strain was sequenced, and the data were subsequently uploaded to the NCBI database (https://www.ncbi.nlm.nih.gov/nuccore/MT496893.1). The strain exhibited a high degree of purity, as demonstrated through fragment analysis of the PCR data, specifically for the target genes of interest.

### IVT and Cas9 cleavage assay

PCR products were amplified from a pBv1-U6-BB plasmid with primers ASFV-sg01-T7IVT-F (ctaatacgactcactataggggcttgcacaggtgtctacat)and pBv1-U6-Filler-XhoI-R, (ctcgagttagcggcatccctgcaagg) to append the T7 promoter at the 5′ end of the multiplex guide RNA cassette. The PCR products were TOPO cloned using pClone007 Versatile Simple Vector Kit (Tsingke) and the TOPO plasmid was isolated. The TOPO plasmid containing the multiplex guide RNA cassette was digested with X*ho* I and purified to be used as an IVT template. IVT was carried out using MEGAscript T7 Kit (Thermo Fisher Scientific) and purified by MEGAclear Kit (Thermo Fisher Scientific) following the manufacturer’s instructions. The purified IVT product was analyzed using 2% agarose gel electrophoresis (Fig. 2a). DNA substrates containing the guide RNA target sites were PCR amplified from purified ASFV genomic DNA using primers detailed in Table S2. *In vitro*, the Cas9 cleavage assay was carried out using purified IVT product and Cas9 protein (Cas9 Nuclease, S. pyogenes, New England BioLabs) following the manufacturer’s instructions. The digested products were analyzed using 2% agarose gel electrophoresis (Fig. 2a).

### Cell culture of pig fibroblast and COS-7 cells

Pig fibroblast cells were isolated from wild-type large white ear samples and cultured in Dulbecco’s modified Eagle’s medium (DMEM; high glucose, GlutaMAX Supplement, pyruvate, Thermo Fisher Scientific) supplemented with 20% fetal bovine serum (Thermo Fisher Scientific), 1% Penicillin/Streptomycin (Pen/Strep, Thermo Fisher Scientific), and 1% HEPES (Sangon). Pig fibroblast cells were maintained in a humidified tri-gas incubator at 37°C and 5% CO_2_, 5% O_2_, and 90% N_2_.

COS-7 cells (CL0075, Hunan Fenghui Biotechnology Co., Ltd) were cultured in Dulbecco’s modified Eagle’s medium (DMEM, high glucose, GlutaMAX Supplement, pyruvate, Thermo Fisher Scientific) supplemented with 10% fetal bovine serum (Thermo Fisher Scientific), 1% Penicillin/Streptomycin (Pen/Strep, Thermo Fisher Scientific), and 1% HEPES (Sangon). COS-7 cells were maintained in a humidified incubator at 37°C with 5% CO_2_.

### Gene editing and single-cell cloning of the pig fibroblast and COS-7 cells

Gene editing of COS-7 cells was performed by electroporation of COS-7 cells with Super PiggyBac plasmid (PB210PA-1, System Biosciences) and one of the five plasmids of pBv1-EF-6X, pBv1-U6-6X, pBv2-EF-6X, pBv2-U6-6X, and pBv1-U6-BB plasmids using Neon Transfection System (Invitrogen). The transfected cells were single-cell sorted using SONY SH800S into 96-well plates, single-cell clones were grown and clones positive for Cas9 gene cassette by PCR were chosen for Cas9 expression analysis. The final clones chosen for analysis are FY10, FY12, GC17, GC49, GE22, GE64, GF16, GF29, and FZ12 (Fig. 1b).

Gene editing of pig fibroblast cells was performed by electroporation of wildtype large white pig fibroblast cells with a plasmid encoding Piggybac transposase and pBv1-EF-6X plasmid using Neon Transfection System (Invitrogen). The edited cells were single-cell sorted using SONY SH800S into 96-well plates, single-cell clones were grown and clones positive for Cas9 gene cassette were chosen for Cas9 expression analysis. Clone number GI58 with an expression of Cas9 was chosen for pig cloning (Fig. 3a).

### Virus inoculation in COS-7 cells *in vitro*

Virus inoculation experiments were standardized according to the Laboratory Biosafety Manual and strictly performed in the P3 biosafety laboratory. The COS-7 cell virus inoculation experiments were carried out in the Animal Biosafety Level-3 Laboratory (ABSL-3) at South China Agricultural University (Guangzhou, China). Wild-type COS-7 cells were used to adapt ASFV to more robust replication after passing ASFV in COS-7 cells for nine generations. The supernatant containing the adapted ASFV virus was harvested from the infected COS-7 at P9 adaption and was used for *in vitro* virus inoculation. Gene-edited COS-7 single-cell clones were seeded into a 24-well plate at 2 × 10^5^ cells/well, 12 wells/clone, and grown in regular media for 1 day before being changed into 500 µL media containing Dulbecco’s modified Eagle’s medium supplemented with 2% fetal bovine serum, 1% Penicillin/Streptomycin, and 1% HEPES. 10 µL of the adapted virus was inoculated into each well of the COS-7 single-cell clones, two wells were harvested each day, and the cells were lysed for further analysis.

### PCR, qPCR, and RT-qPCR

PCR for the presence of Cas9 transgene was performed using the following primers: FzCas9-yg-R3, gcatgaagtttctgttggcgaag; FzCas9-yg-F4, cctatgcccacctgttcgac*.* Cycling conditions were as follows: 95℃ for 3 min; 30 cycles of 95℃ for 15 s, 60℃ for 15 s, and 72℃ for 15 s; 72℃ for 5 min.

qPCR for ASFV quantification was performed with the following primers: ASFV-Tqm-Pb1x, ccacgggaggaataccaacccagtg; ASFV-Tqm-Fw12, gatgatgattaccttygctttgaa; ASFV-Tqm-Rv12, tctcttgctcrtrgatacrttaatatga. Cycling conditions were as follows: 95℃ for 3 min; 40 cycles of 95℃ for 30 s and 60℃ for 1 min.

RT-qPCR for quantification of Cas9 RNA expression level was performed using the following primers: fzCas9-yg-R2: tctggtcatccaggcgaatc; fzCas9-yg-F3, gaccttcgacaacggcagcatc. Cycling conditions were as follows: 95℃ for 2 min; 45 cycles of 95℃ for 20 s, 60℃ for 34 s, and 72℃ for 34 s, with cDNA reverse-transcribed from isolated total RNA from corresponding cells.

### Somatic cell microinjection to produce SCNT embryos and embryo transfer for pig cloning

Animal experiments involving ASFV were standardized according to the Laboratory Biosafety Manual and strictly performed in the P3 biosafety laboratory. The animal experiments were carried out in the Animal Biosafety Level-3 Laboratory (ABSL-3) of the Spirit Jinyu Biological Pharmaceutical Co., LTD (Neimenggu, China). The somatic cell microinjection procedure and SCNT were conducted as previously described (21, 24–26).

### Virus source, pig background, and virus challenge in gene-edited pigs *in vivo*

ASFV was isolated from clinical samples and identified as genotype II strain GZ201801 by the sequence of p72 protein (GenBank ID: MT496893.1). In all, 17 large white pigs (female, 70–82 days old) were enrolled in the experiment including 5 transgenic and 12 wild-type pigs. In total, 14 animals were randomly set into two groups and challenged with ASFV. The group setting and challenge route are shown in Table 1 .

**TABLE 1:** Experimental animal grouping and challenge^*a*^

Groups	Number of animals (head）	Challenge route	Challenge dosage
Challenge group of transgenic pigs	3 (transgenic pigs challenge) + 3 (wild-type pigs cohabit) + 1 (transgenic pigs cohabit)	Intramuscular injection	10^3^ HAD_50_/mL 2 mL
Challenge group of wild-type pigs	3 (wild-type pigs challenge) +3 (wild-type pigs cohabit) +1 (transgenic pigs cohabit)	Intramuscular injection	10^3^ HAD_50_/mL 2 mL
Wild-type pigs (negative)	3	—	—

^*a*^ Due to the limited number of transgenic pigs, they were arranged into two groups to test the resistance to direct challenge and cohabit challenge.

The challenge experimental groups and the non-challenge negative group were fed by different workers and housed in isolated rooms to prevent cross-infection.

Before the experiment, all animals were ear-tagged and tested negative for ASFV antigens and antibodies. Three days before the challenge, EDTA anticoagulated blood and whole blood, as well as oropharyngeal and anal swabs were collected from all animals. Clinical symptom scores and temperature (rectal) of animals were recorded daily. Clinical symptoms were evaluated by the caretakers according to Table 2.

**TABLE 2:** Clinical symptom index scoring criteria

Observation item	Scoring criteria	Score
a Appetite	Normal Slight loss Significant loss Complete loss	0 1 2 3
b Mental state	Normal Slow movement and low spirits Often get down and depressed Lie on the ground and drowsy	0 1 2 3
c Temperature (rectal temperature）	Normal (38°C - 40°C) Low fever (40°C - 42°C) High fever (>42°C)	0 1 3
Overall clinical score = a + b + c		

Animals were challenged on day 0. For all experimental animals, oropharyngeal swabs and anal swabs were collected daily from day 0 of the experiment. Besides, EDTA anticoagulated blood and whole blood were collected every 2 days from day 1 until the end of the experiment or the death of animals. During the experiment, dying animals were euthanized and dissected for sampling. The experiment ended on day 15. All remaining animals were euthanized and then dissected for sampling.

ASFV antigens were tested in EDTA anticoagulated blood samples and swab samples through real-time PCR. During the necropsy, various tissues and organs were harvested, and the presence of ASFV antigens in these fresh samples was assessed using real-time PCR (Table S3).

## AFTER EPUB

*[This article was published on 2 April 2024 with errors in the byline order. The byline was corrected in the current version, posted on 13 June 2024.]*

## SUPPLEMENTAL MATERIAL

Tables S1 spectrum.02164-23-s0001.TIF Supplemental table.

Tables S2 spectrum.02164-23-s0002.TIF Supplemental table.

Tables S3 spectrum.02164-23-s0003.TIF Supplemental table.

Tables S4 spectrum.02164-23-s0004.TIF Supplemental table.

Fig. S1 spectrum.02164-23-s0005.TIF Supplemental figure.

Fig. S2 spectrum.02164-23-s0006.TIF Supplemental figure.

ASM does not own the copyrights to Supplemental Material that may be linked to, or accessed through, an article. The authors have granted ASM a non-exclusive, world-wide license to publish the Supplemental Material files. Please contact the corresponding author directly for reuse.
