# Supplementary figures and images for: Testing multiplexed anti-ASFV CRISPR-Cas9 in reducing African swine fever virus
Source: Microbiol Spectr. 2024 Apr 2;12(7):e02164-23. doi: 10.1128/spectrum.02164-23 (PMC11218517; doi:10.1128/spectrum.02164-23)

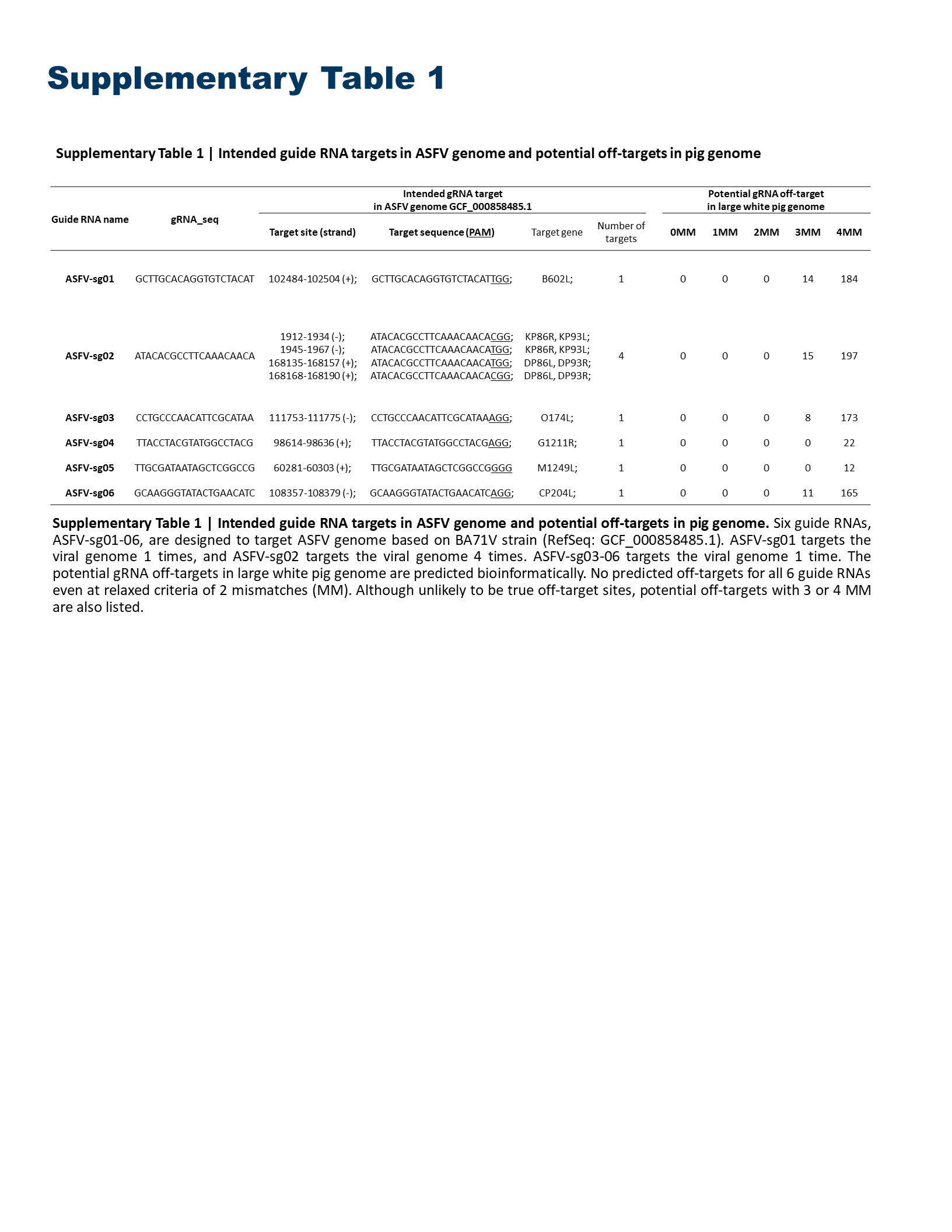

Supplement: Tables S1 — Supplemental table. [file spectrum.02164-23-s0001.tif]

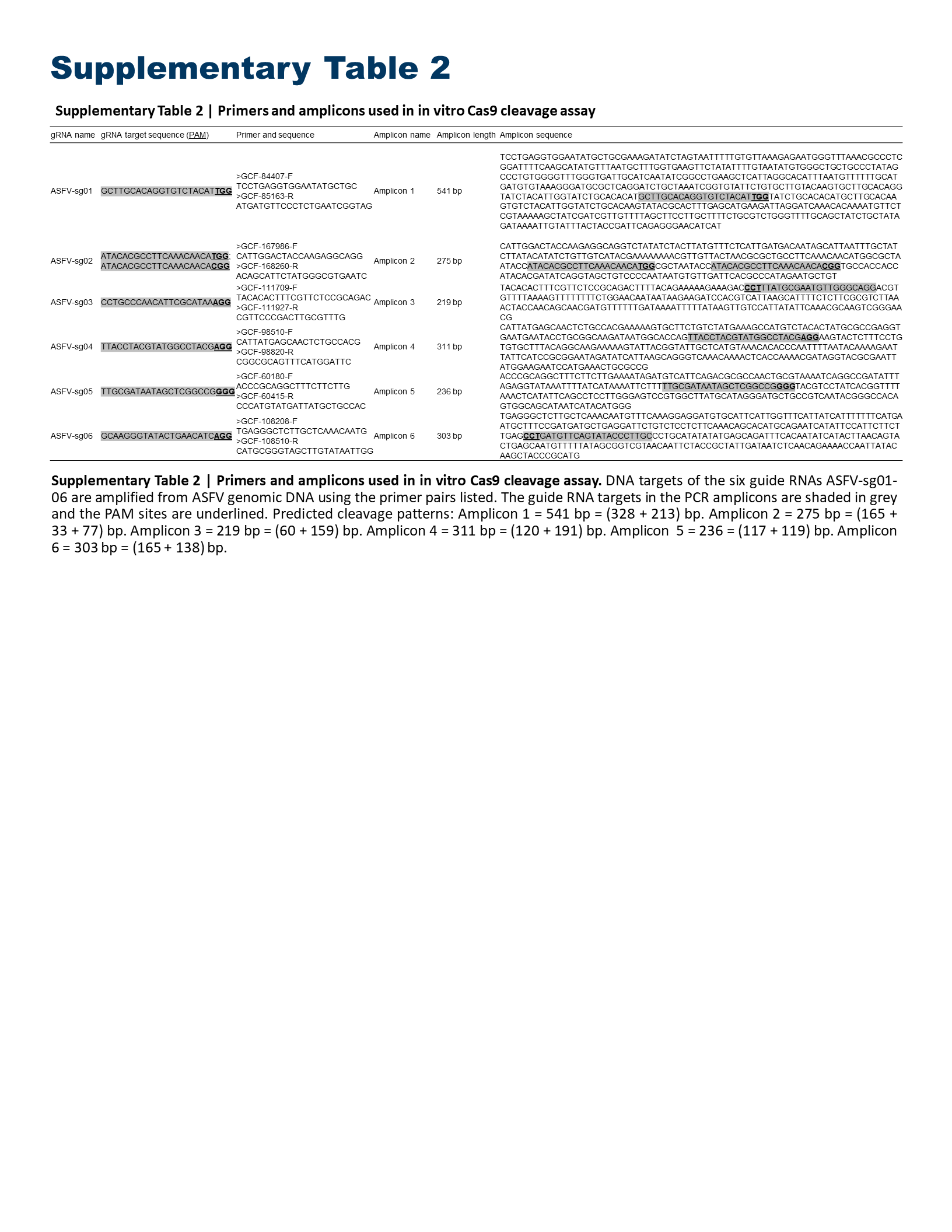

Supplement: Tables S2 — Supplemental table. [file spectrum.02164-23-s0002.tif]

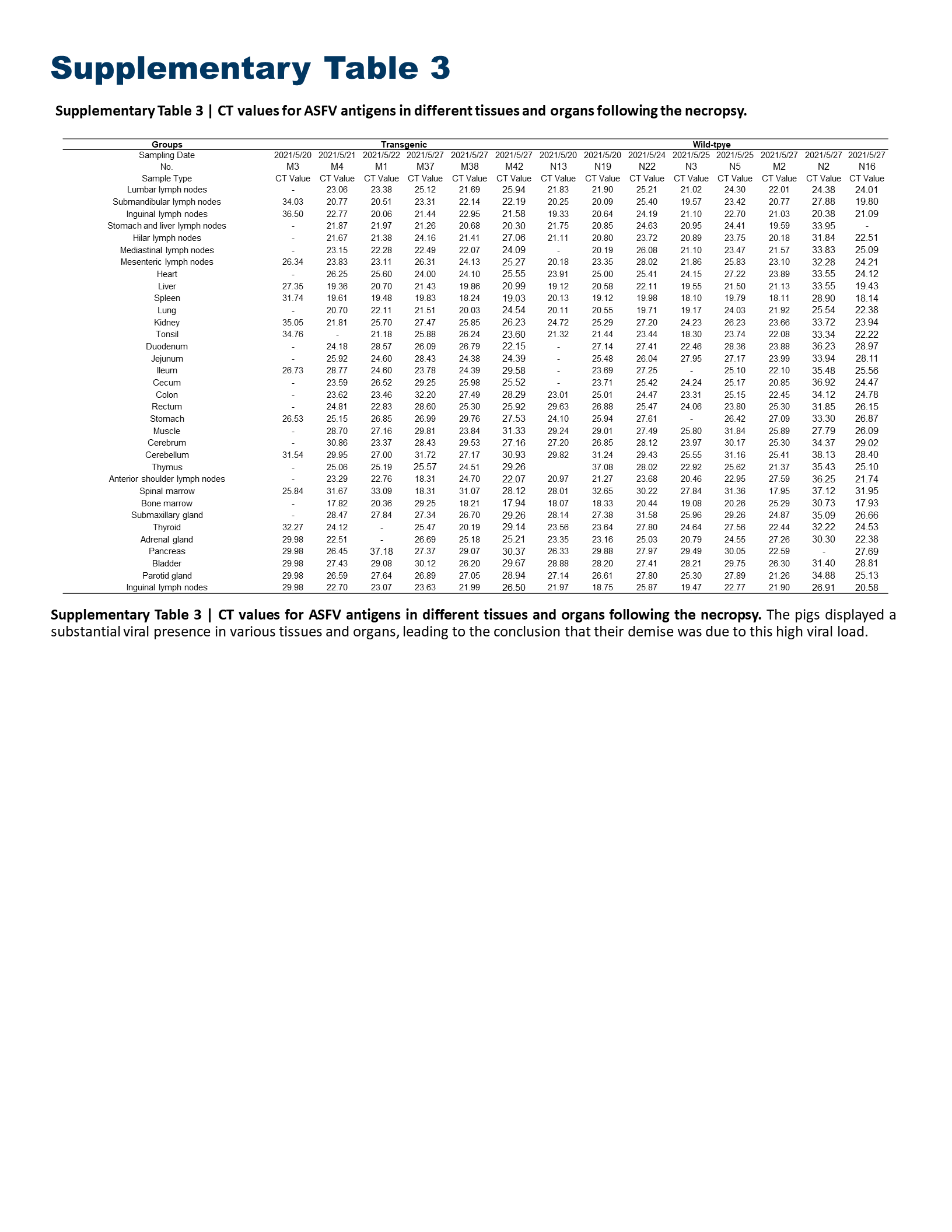

Supplement: Tables S3 — Supplemental table. [file spectrum.02164-23-s0003.tif]

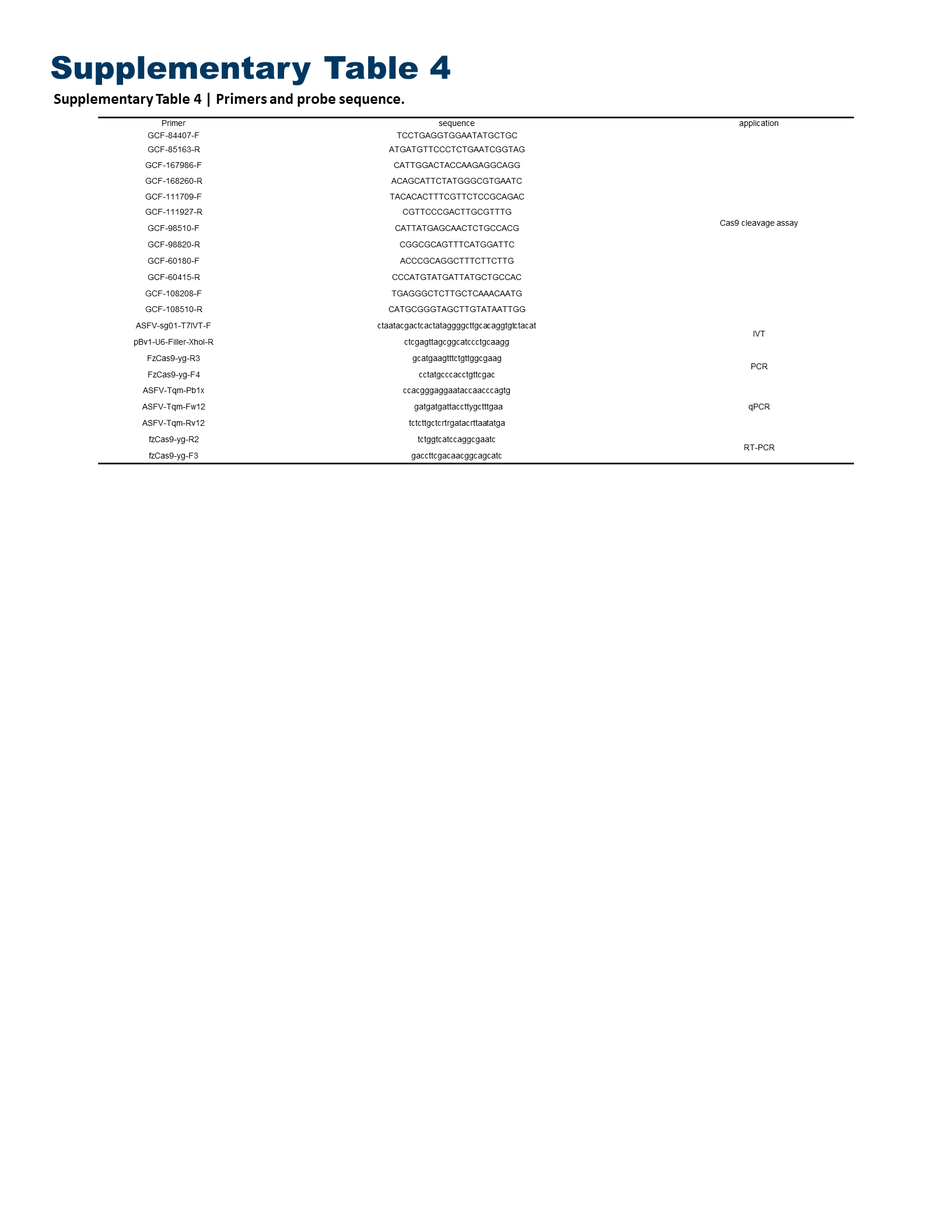

Supplement: Tables S4 — Supplemental table. [file spectrum.02164-23-s0004.tif]

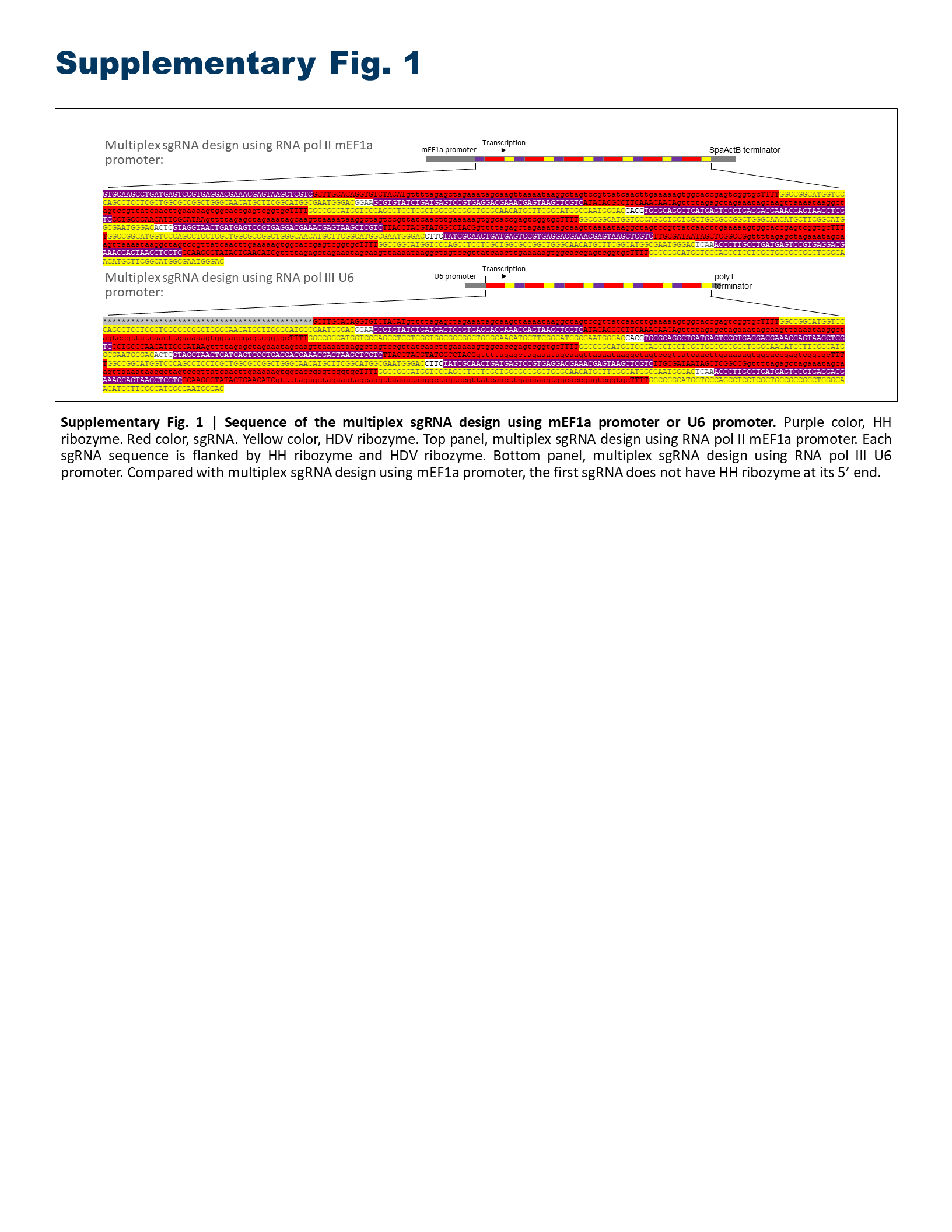

Supplement: Fig. S1 — Supplemental figure. [file spectrum.02164-23-s0005.tif]

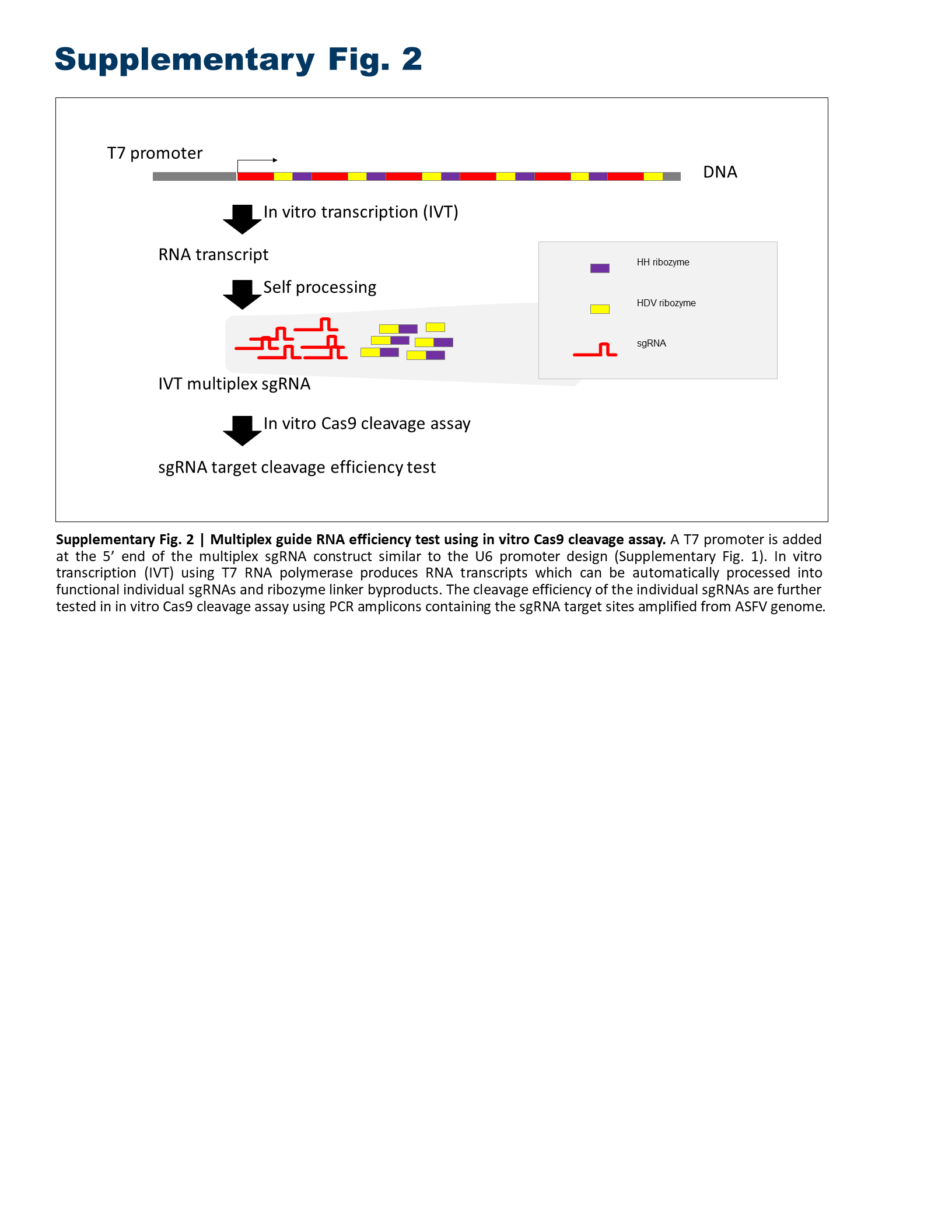

Supplement: Fig. S2 — Supplemental figure. [file spectrum.02164-23-s0006.tif]
